# Cognitive Tendencies Influencing Decision-Making in Young Footballers and the Role of Psychological Support

**DOI:** 10.3390/bs16020205

**Published:** 2026-01-30

**Authors:** Mehmet Kara, Murat Genç, Laurentiu-Gabriel Talaghir, Cristina Corina Bențea, Bogdan Sorin Olaru, Paula Ivan

**Affiliations:** 1 Faculty of Sport Sciences, Mersin University, Mersin 33343, Turkey; mehmetkara@mersin.edu.tr; 2 Faculty of Physical Education and Sport, “Dunărea de Jos” University of Galați, 800008 Galați, Romania; bogdan.olaru@ugal.ro (B.S.O.); paula.ivan@ugal.ro (P.I.); 3 Faculty of Education Science, “Dunărea de Jos” University of Galați, 800008 Galați, Romania; cristina.bentea@ugal.ro

**Keywords:** automatic thoughts, dysfunctional attitudes, effective decision making, moderated mediation, sports psychologist

## Abstract

Effective decision-making is critical in high-performance sports like football and is heavily influenced by cognitive processes. This study applies Beck’s Cognitive Theory to investigate the mechanisms through which automatic thoughts impact decision-making in young footballers, and how psychological support might alter this relationship. The primary objective was to test a model where dysfunctional attitudes mediate this process, and sports psychologist support acts as a moderator. A correlational survey was conducted with 636 actively licensed footballers (18–30 years old) from Turkey. Data were collected using the Automatic Thoughts Questionnaire (ATQ), the Dysfunctional Attitude Scale (DAS), and the Scale of Effective Decision-Making in Sport (SEDMS). A moderated mediation model was employed to analyze the direct, indirect, and conditional effects of the variables. The findings revealed that dysfunctional attitudes partially mediated the negative relationship between automatic thoughts and effective decision-making (indirect effect = −0.043, 95% CI [−0.077, −0.012]), accounting for 16.8% of the total effect. Furthermore, sports psychologist support significantly moderated the direct pathway between automatic thoughts and decision-making (interaction β = 0.312, *p* = 0.002). Simple slope analysis showed that the detrimental impact of automatic thoughts on decision-making was substantially weaker for athletes who had received psychological support (β = −0.142) compared to those who had not (β = −0.353). Automatic thoughts impair footballers’ decision-making, a process that is significantly explained by the activation of underlying dysfunctional attitudes. Professional psychological support serves as a critical cognitive buffer, enhancing athletes’ cognitive resilience against these negative thought patterns. The findings provide an evidence-based justification for integrating sport psychologists into athletic programs to foster better on-field performance.

## 1. Introduction

Effective decision-making in sports transcends mere technical skills, involving a complex interplay of cognitive and emotional factors that are vital under pressure (Chen et al., 2023; Wu et al., 2021). The contemporary understanding of sports performance recognizes that it is shaped by cognitive and emotional components and physical capabilities (Vealey & Chase, 2016). Among these critical cognitive components, decision making emerges as a determinant of game strategy, particularly in disciplines requiring speed (Eccles, 2020; Farrow et al., 2013). Indeed, the quality of decisions is directly linked to the cognitive resilience exhibited under the stress load of the sporting environment (Kahneman, 2011; Sweller, 2011). Cognitive resilience can be defined as the mental capacity to effectively manage and adapt one’s cognitive processes under stress, maintaining optimal decision-making and emotional regulation despite challenging circumstances (Balconi & Rovelli, 2024; Thomas & Zolkoski, 2020). The cognitive patterns of athletes, including their automatic thoughts, significantly impact their performance and psychological resilience under competitive pressure (Mojtahedi et al., 2023; Raab, 2012). It is, therefore, essential to understand these fundamental aspects.

One of the primary elements threatening cognitive resilience is the frequency of automatic thoughts (A. T. Beck, 1995; Netemeyer et al., 2002), which is defined as the intensity of involuntary and often dysfunctional cognitions emerging in an individual’s mind. According to Beck’s Cognitive Theory (A. T. Beck, 1979), these momentary thoughts do not exist in isolation; they both feed and are fed by dysfunctional attitudes, rigid and unrealistic belief systems. Despite its widespread use in clinical settings, Beck’s cognitive theory (A. T. Beck, 1979) has not been fully leveraged in sports psychology, which holds significant potential. Recent findings suggest that athletes’ attentional control and decision-making can be adversely affected by dysfunctional attitudes and negative automatic thoughts (Gardner & Moore, 2021). These attitudes are cognitive schemas containing excessive beliefs about issues such as success or approval (Batmaz & Özdel, 2016; Weissman & Beck, 1978) and are reported to cause internal conflict when athletes make decisions (J. S. Beck, 2011; Field, 2024). Thus, it is hypothesized that the frequency of automatic thoughts negatively impacts cognitive performance, an effect that is potentially amplified by an individual’s pre-existing dysfunctional attitudes (Kwon & Oei, 1994). Originally developed within clinical psychology to assess cognitive vulnerabilities associated with depression (A. T. Beck, 1979; Weissman & Beck, 1978), these constructs hold significant relevance in high-stress environments like competitive sports. Recent research has highlighted that athletes, despite perceptions of high mental fortitude, are susceptible to depressive symptoms, which can directly impair cognitive functions crucial for performance, such as attention and decision-making (Gouttebarge et al., 2015; Rice et al., 2016).

However, these cognitive mechanisms are not independent of the external support systems available to athletes. From a cognitive–behavioral perspective, professional psychological support has been observed to positively intervene in an individual’s automatic thought patterns through cognitive restructuring (Arvinen-Barrow & Walker, 2013; J. S. Beck, 2011). Sports psychologist support may, thus, serve as a critical external factor that enhances athletes’ cognitive resilience. Psychological support is not only associated with clinical issues but also directly related to performance-oriented cognitive development (Andersen, 2000), and it may assume a regulatory (moderating) role by buffering the adverse effects of this cognitive cycle.

Although the connection between automatic thoughts and decision-making is well established, the specific pathways of this relationship and the conditions under which it might be mitigated remain empirically unexplored in a sports context. The role of dysfunctional attitudes as mediating factors has not yet been systematically tested. Existing research has generally focused on direct effects or failed to integrate mediating and moderating variables within a single model. Thus, a significant gap persists, particularly concerning how this mediating role might differ according to athletes’ career stage (Hayes, 2017; MacKinnon et al., 2007). The 18–30 age range is of critical importance in this context, as this period is defined as the stage where both physical and cognitive performance peak and professional pressures are most intensely experienced (Arvinen-Barrow & Walker, 2013; Vealey & Chase, 2016).

The contributions of this research to the literature are fourfold: (1) it empirically tests the role of dysfunctional attitudes as a mediating factor in the relationship between automatic thoughts and decision-making; (2) it examines this model in a high-cognitive-demand field, namely football; (3) it carries out this analysis on athletes within the 18–30 age range, who are at the peak of their careers; and (4) it provides a methodological contribution by simultaneously testing both mediation and moderation effects within a single integrated model. In line with this theoretical framework, the primary aim of this research is to examine, within a holistic model, the mediating role of dysfunctional attitudes in the effect of automatic thought frequency on effective decision-making in sports, and the moderating effect of sports psychologist support on this relationship. Thus, the following hypotheses were tested:

**H1.**  *Automatic thought frequency significantly and negatively predicts the level of effective decision-making in sports*.

**H2.**  *Automatic thought frequency significantly and positively predicts dysfunctional attitudes*.

**H3.**  *Dysfunctional attitudes significantly and negatively predict effective decision-making in sports*.

**H4.**  *Dysfunctional attitudes play a significant mediating role in the relationship between automatic thought frequency and effective decision making in sports*.

**H5.**  *Sports psychologist support has a moderating effect on the relationship between automatic thought frequency and effective decision making in sports*.

## 2. Materials and Methods

This section presents detailed information regarding the population, sample, data collection instruments, research process, and analytical techniques employed in the study.

### 2.1. Research Model

**Figure 1:**
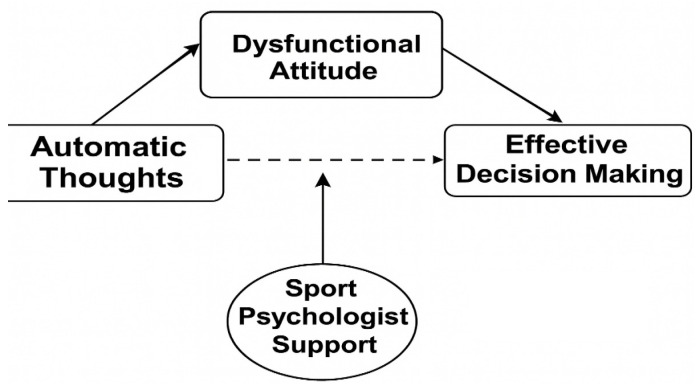
Conceptual model illustrating the mediating role of Dysfunctional Attitudes and the moderating role of Sports Psychologist Support in the relationship between Automatic Thought Frequency and Effective Decision Making in Sports.

This study employed a predictive correlational design to examine the non-causal relationships between variables and evaluate the predictive effects of certain variables on others, which allows for a holistic examination of the direct, indirect, and moderating effects tested, thereby enabling a comprehensive evaluation of the interaction between cognitive tendencies and environmental support in footballers’ decision-making processes. The research employed convenience sampling, a non-probability method chosen for its practicality in accessing a wide-ranging and geographically diverse group of active football players (Robbins et al., 2021; Tyrer & Heyman, 2016). Data were collected in September 2025 from licensed male and female athletes in various regions of Turkey. The research model was structured within the framework of Beck’s Cognitive Theory (A. T. Beck, 1995), testing the mediating role of dysfunctional attitudes and the moderating role of sports psychologist support in the relationship between automatic thought frequency and effective decision-making in sports. The conceptual model developed in this study is illustrated in Figure 1.

### 2.2. Ethical Considerations

This study was conducted in accordance with the ethical principles and standards set forth in the 2008 Declaration of Helsinki. Ethical approval for the research was obtained from the Mersin University Sports Sciences Ethics Committee (date: 1 September 2025, Decision No: 2025-71). The purpose of the research was clearly explained to the participants, and their participation was voluntary. Data were collected anonymously between 3 and 30 September 2025, and were used solely for scientific purposes. Additionally, AI-assisted tools have been employed for technical purposes, such as language refinement, proofreading, and reference formatting. These tools were used solely to enhance clarity and presentation and did not influence the study’s original content, analyses, or scientific conclusions.

### 2.3. Participants

Data were obtained from 672 active footballers who voluntarily participated; however, following analyses of statistical assumptions, the study was performed with 636 observations. Of the participants, 44.5% were female (n = 283) and 55.5% were male (n = 353). This distribution permits a comparative interpretation of the findings regarding both female and male footballers. Regarding age distribution, the vast majority (72.5%) were footballers in the 18–24 age range (n = 461), whereas 27.5% were in the 25–30 age range (n = 175). Regarding football experience, 29.1% of the study group had 1–3 years (n = 185), 23.7% had 4–6 years (n = 151), 27.4% had 7–10 years (n = 174), and 19.8% had 11 or more years (n = 126) of experience as licensed players. Additionally, 26.4% of footballers (n = 168) reported having sustained a serious injury in the past, whereas 73.6% (n = 468) reported no such injury. The distribution of footballers by position showed that 14% were goalkeepers (n = 89), 30.8% were defenders (n = 196), 36% were midfielders (n = 229), and 19.2% were forwards (n = 122), which allows for the findings to be discussed in the context of on-field positions. Regarding psychological support, 44.49% of the footballers (n = 283) reported having received support from a sports psychologist. In the context of this study, this support was defined as having had at least one formal session with a certified sports psychologist, either affiliated with their club or privately, focusing on performance-related issues such as competition anxiety, focus, and motivation. The remaining 55.51% (n = 353) reported no such support. It is important to note that the nature, frequency, and duration of this support were not standardized across participants, a point that is addressed as a limitation of the study.

### 2.4. Data Collection

In addition to collecting demographic information, such as participants’ gender, age, years of licensed football experience, history of serious injury, playing position, and whether they had received support from a sports psychologist, data were obtained through three distinct measurement instruments.

#### 2.4.1. Automatic Thoughts Questionnaire (ATQ) Short Form

The study utilized the 8-item short-form version of the Automatic Thoughts Questionnaire originally developed by Netemeyer et al. (2002). Gökdağ and Kaçar-Başaran (2019) adapted this short form to Turkish culture. The scale consists of a single-factor structure comprising eight items rated on a 5-point Likert-type scale. Confirmatory factor analysis results revealed that this structure was unidimensional and demonstrated a good model fit (CFI = 1.00, NNFI = 1.00, RMSEA = 0.037–0.071). The scale’s internal consistency coefficient demonstrated high reliability with Cronbach’s α = 0.91 (Gökdağ & Kaçar-Başaran, 2019). In the current study, Cronbach’s α was calculated as 0.80, confirming a high level of reliability.

#### 2.4.2. Dysfunctional Attitude Scale (DAS)

Developed by Weissman and Beck (1978) and adapted to Turkish culture by Batmaz and Özdel (2016), this scale consists of two subscales: “Perfectionism/Achievement” and “Need for Approval/Dependency.” The instrument comprises 13 items measured on a 7-point rating scale ranging from 1 (strongly disagree) to 7 (strongly agree). Higher scores indicate higher levels of dysfunctional attitudes. Reliability analyses yielded Cronbach’s α = 0.84 (Batmaz & Özdel, 2016). In the present study, the total scale score was used, and the reliability coefficient was determined as Cronbach’s α = 0.90.

#### 2.4.3. Scale of Effective Decision-Making in Sport (SEDMS)

Developed by Çetin and Kara (2024), this scale measures athletes’ effective decision-making processes through a two-dimensional structure consisting of 15 items: Internal Decision-Making (n = 7) measures the athlete’s decisions based on internal processes, scored normally, while External Decision-Making (n = 8) measures decisions influenced by external factors. A 5-point Likert-type rating scale was used: 1 (strongly disagree) to 5 (strongly agree). A score of 1 (strongly disagree) indicated low competence, while a score of 5 (strongly agree) indicated high competence. Analyses conducted to determine the scale’s structural validity and reliability yielded Cronbach’s alpha internal consistency coefficients of 0.87 for the “External Decision-Making” subscale and 0.85 for the “Internal Decision-Making” subscale. The model fit indices were CFI = 0.96 and NNFI = 0.96. These findings indicate a high inter-item consistency and support the reliability of the measurement instrument. In alignment with the study’s concentration on personal cognitive mechanisms, as articulated by Beck’s theory (A. T. Beck, 1995), the research exclusively utilized the Internal Decision-Making subscale, which demonstrated a Cronbach’s α of 0.87. The data obtained from these instruments were subjected to statistical analyses following the necessary assumptions.

### 2.5. Data Analysis

During data collection, actively licensed footballers aged 18–30 years were recruited, and data were obtained via an online survey method. The resulting observations were initially inspected for missing data, outliers, and fulfillment of the assumptions required for the statistical analysis. The online collection method yielded no missing data. Following preliminary checks and outlier analysis, 636 valid observations were deemed suitable for analysis.

Prior to analysis, the dataset consisting of 672 observations was transferred to a statistical software, and the normality assumption was evaluated through central tendency measures, including mode, median, and arithmetic mean. The proximity observed among these three measures indicates that the distribution exhibits normal characteristics. Prior to analysis, the data were screened for missing values and univariate and multivariate outliers using Z-scores and Mahalanobis distance, respectively (Tabachnick & Fidell, 2013). This screening process removed 36 observations, resulting in a final sample of 636 participants for the main analysis.

The Durbin-Watson test was applied to assess whether autocorrelation existed among the error terms in the dataset. The obtained Durbin-Watson coefficient was 1.204, indicating that the error terms were independent of each other and that no autocorrelation was present (Kalaycı, 2010). To evaluate the multicollinearity problem, the Variance Inflation Factor (VIF) and tolerance values were examined. The analyses revealed that the VIF values ranged from 1.602 to 3.036, and the tolerance values ranged from 0.347 to 0.604. All tolerance values were above 0.20, and VIF values below five indicated that there was no multicollinearity problem in the model (Makrakis et al., 2024; Raheem et al., 2019). All assumption tests and data analyses were conducted using Jamovi 2.6.2. In addition, the suitability of the dataset for parametric analyses was comprehensively evaluated in terms of normality, outliers, and multicollinearity. The findings regarding the assumption of normality are presented in Table 1.

Kolmogorov–Smirnov and Shapiro–Wilk normality tests were applied to determine the distribution properties of the scores from the three measurement instruments (ATQ, DAS, and SEDMS). The results indicated that all three variables deviated from a normal distribution at a statistically significant level (*p* < 0.001 for Kolmogorov–Smirnov tests; *p* < 0.001 for Shapiro–Wilk tests) (Field, 2024). This was observed for the ATQ (K-S(636) = 0.136, *p* < 0.001; S-W = 0.927, *p* < 0.001), the DAS (K-S(636) = 0.083, *p* < 0.001; S-W = 0.973, *p* < 0.001), and the SEDMS (K-S(636) = 0.091, *p* < 0.001; S-W = 0.957, *p* < 0.001).

Nonetheless, owing to the heightened sensitivity of these tests in large sample sizes (n > 300), even minor discrepancies can result in statistically significant findings that may not have substantial practical relevance (Tabachnick & Fidell, 2013). Thus, relying solely on these test outcomes is insufficient to assess normality. To provide a more robust evaluation, it is recommended that supplementary statistical criteria that reflect the shape of the distribution be examined. In this context, skewness values and their derived z-scores were calculated. According to the criteria proposed by Kim (2013), z-skewness values within the ±1.96 range are accepted as a strong indicator of normal distribution, an additional analysis that provides a more comprehensive and reliable assessment of the normality assumption. Skewness values were also examined and are presented in Table 2.

According to the data in Table 2, the skewness coefficients were 0.878 for the DAS, 0.239 for the ATQ, and −0.510 for the SEDMS; the standard error for all variables was 0.97. The standardized z-scores calculated from these values were 0.905 for the DAS, 0.246 for the ATQ, and −0.525 for the SEDMS. The observation that all z-scores fell within the +/− 1.96 limits indicates that the distributions demonstrate near-normal characteristics (Kim, 2013). In view of these values, the dataset was deemed suitable for parametric statistical analysis, and confirmatory factor analysis (CFA) was subsequently performed. Table 3 presents the CFA results.

The CFA findings reported in Table 3 demonstrate that the model fit levels for all three measurement instruments were generally within acceptable limits. For the DAS, the chi-square/degrees of freedom ratio (χ^2^/df = 2.45), along with CFI = 0.89, TLI = 0.88, SRMR = 0.05, and RMSEA = 0.08, indicates that the model possesses adequate, albeit limited, suitability. The fit indices obtained for the ATQ (χ^2^/df = 3.67, CFI = 0.93; TLI = 0.90; SRMR = 0.06; RMSEA = 0.08) suggest a good level of model fit. Similarly, the values calculated for the SEDMS (χ^2^/df = 3.14, CFI = 0.92, TLI = 0.89, SRMR = 0.04, RMSEA = 0.07) show that this scale offers an acceptable level of structural validity. Thus, based on confirmatory factor analyses, it can be stated that all three measurement instruments employed in the study exhibit satisfactory model fit and that their measurement structures are statistically valid and reliable (Hu & Bentler, 1999; Kline, 2023).

In this study, generative artificial intelligence was utilized in the data visualization process. Specifically, the free version of ChatGPT (based on the GPT-3.5 model) was used to assist in the creation of the moderation plot. The authors reviewed the generated visual output for accuracy and clarity, taking full responsibility for the final representation of the data.

## 3. Results

This section statistically examines the relationships between the variables included in the model. A fundamental assumption of the mediation analysis is the presence of significant relationships among the constructs within the model. Within this framework, statistically significant correlations between the independent and dependent variables, the independent and mediator variables, and the mediator and dependent variables are prerequisites for testing the mediation effect (Baron & Kenny, 1986). In accordance with this assumption, the relationships among the variables were evaluated using Pearson’s correlation coefficients prior to the main analysis. These findings support the directional and significant relationships posited in the model. The correlation values are presented in Table 4.

According to the correlation analysis in Table 4, statistically significant relationships were found between the study variables. A positive, moderate, and significant relationship was observed between DAS and ATQ (r = 0.422, *p* < 0.01), a finding that supports H2 and indicates that individuals’ dysfunctional attitudes tend to increase in line with their level of automatic thoughts. Conversely, a negative, low-level significant relationship was found between DAS and SEDMS (r = −0.205, *p* < 0.01), which supports the prerequisites for H3. This finding offers initial evidence in support of H3, indicating that inflexible belief systems may hinder effective decision making within the context of gameplay. Similarly, a significant and negative relationship was identified between the ATQ and SEDMS (r = −0.273, *p* < 0.01). H1 was therefore supported, showing that ATQ significantly and negatively predicted the level of effective decision-making. This analysis suggests that individuals may be less effective in their decision-making processes when they resort to automatic thoughts more frequently. According to Cohen’s (2013) classification, correlation coefficients of 0.10–0.29 indicate a low effect, 0.30–0.49 a medium effect, and ≥0.50 a high effect. In this context, the findings satisfied the relational prerequisites necessary for testing the mediation model.

Additionally, the reliability levels of the instruments were assessed using Cronbach’s Alpha coefficients, which were 0.90 for the DAS, 0.80 for the ATQ, and 0.87 for the SEDMS. These values show that the scales have a high level of internal consistency (Field, 2024; George & Mallery, 2024). Alpha coefficients above 0.80 support the assertion that the measurement reliability of the scales is sufficiently high (Tavakol & Dennick, 2011). These significant relationships provide the necessary prerequisites for performing mediation and moderation analyses (Baron & Kenny, 1986), offering a strong foundation for testing the structural model.

### 3.1. Findings Related to the Mediation Model

**Figure 2:**
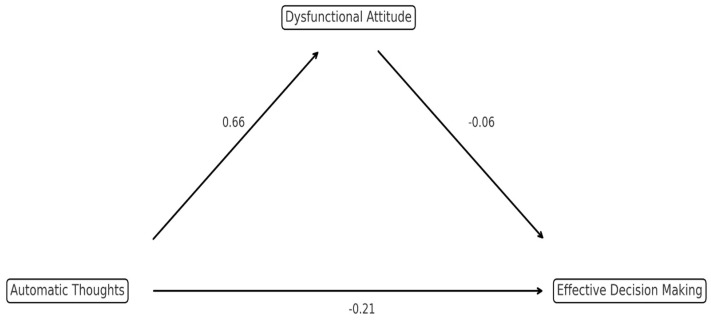
Mediation Model.

Figure 2 presents the structural model, which illustrates the indirect effect of automatic thought frequency (independent variable) on effective decision making in sports (dependent variable) via dysfunctional attitudes (mediator variable). The path coefficients in the model represent the direction and magnitude of the direct relationships between variables.

According to the mediation analysis results (Table 5), the dysfunctional attitude variable played a significant partial mediating role in the effect of the automatic thought frequency variable on effective decision-making in sports.

As shown in Table 5, the path coefficient between automatic thoughts and dysfunctional attitudes was significant and positive (β = 0.6594, Z = 11.75, *p* < 0.001), which indicates that footballers’ tendencies to exhibit dysfunctional attitudes increase as their levels of automatic thought frequency increase. Similarly, a negative and statistically significant relationship was identified between dysfunctional attitudes and effective decision-making (β = −0.0647, Z = −2.59, *p* = 0.010), which suggests that an increase in dysfunctional attitudes may have adverse effects on decision-making skills. The direct effect observed between automatic thoughts and effective decision making in sports was also statistically significant and negative (β = −0.2117, Z = −5.43, *p* < 0.001).

The indirect effect (a × b = −0.0427, Z = −2.53, *p* = 0.011) was also statistically significant, which supports the mediating role of the dysfunctional attitude variable (Baron & Kenny, 1986). Thus, the analysis confirms H4, showing that dysfunctional attitudes play a significant mediating role in this relationship. This indirect effect, which accounted for 16.8% of the total relationship, statistically supported H4. The remaining 83.2% was attributed to the direct effect of automatic thoughts (Hayes, 2017). This confirms that, while automatic thoughts have a strong direct effect, a significant portion of their impact is channeled indirectly through the activation of dysfunctional attitudes.

The total effect of the model was also significant and negative (c + ab = −0.2544, Z = −7.17, *p* < 0.001) (MacKinnon et al., 2007). These findings demonstrate that the effect of automatic thought frequency on effective decision-making in sports can be explained through both direct and indirect pathways and that dysfunctional attitudes function as a significant, albeit limited, mediator in this process. The effect size coefficients for the mediation model are presented in Table 6. The explained variance ratios and effect sizes were examined to evaluate the model’s practical significance. These results show that footballers’ decision-making processes are shaped not only by momentary thoughts but also by deep cognitive schemas.

The findings presented in Table 6 show the explained variance ratios (R^2^) for the dependent variables in the structural model and the corresponding Cohen’s f^2^ effect size. The predictive effect of automatic thought frequency on dysfunctional attitudes accounted for 18% of the variance (R^2^ = 0.180), with Cohen’s f^2^ = 0.22, indicating a medium effect size. The model predicting effective decision-making in sports from both automatic thought frequency and dysfunctional attitude explained 22.9% of the variance (R^2^ = 0.229), with Cohen’s f^2^ = 0.297, representing a substantial effect size. The effect size interpretations were based on Cohen’s (2013) classification guidelines. The assessment of effect sizes in structural models is particularly crucial for establishing both the theoretical validity and practical implications of research findings (Hair et al., 2014; Kline, 2023).

### 3.2. Findings Related to the Moderation Model

**Figure 3:**
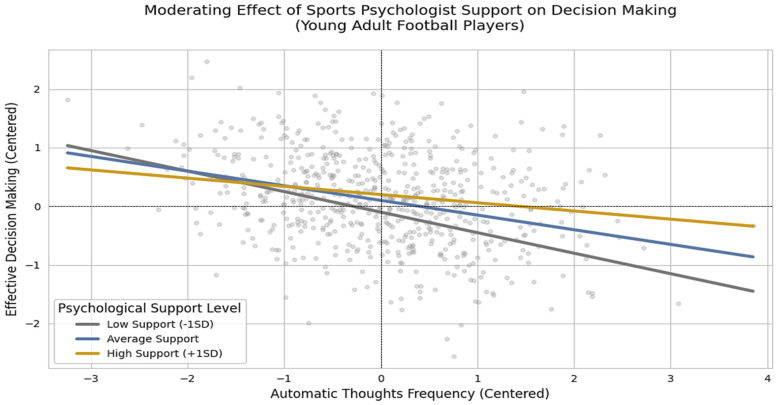
Variation in the effect of automatic thought frequency on effective decision-making in sport according to the level of sports psychologist support.

The moderator model illustrating the variation in the relationship between automatic thought frequency and effective decision-making in sports according to the level of sports psychologist support is shown in Figure 3. Sports psychologist support was categorized by participant responses as “yes, I have previously received support” or “no, I have not previously received support.”

The results of the moderation analysis, presented in Table 7, indicated that receiving sports psychologist support functioned as a significant moderator variable in the relationship between automatic thought frequency and effective decision making in sports. Examination of the coefficients indicated that the direct effect of automatic thought frequency on effective decision making in sports was significant and negative (β = −0.248, SE = 0.0351, Z = −7.06, *p* < 0.001). The direct effect of sports psychologist support was also negative and significant (β = −0.182, SE = 0.0762, Z = −2.39, *p* = 0.017). Crucially, the interaction term is statistically significant (β = 0.312, SE = 0.1001, Z = 3.12, *p* = 0.002). This result demonstrates that footballers’ receipt of sports psychologist support significantly influences the relationship between automatic thought frequency and effective decision-making. Based on these findings, Hypothesis 5 was supported, indicating that sports psychologist support plays a moderating role in the relationship between automatic thought frequency and effective decision-making. This interaction was statistically significant (β = 0.312, *p* = 0.002; ΔR^2^ = 0.06, *p* < 0.01), and the detrimental effect of automatic thought frequency on decision making was attenuated among footballers receiving sports psychologist support.

Simple slope analysis results showed a significant difference in the negative effect of automatic thought frequency on effective decision making according to psychological support status. Among footballers who did not receive psychological support, the negative association between automatic thought frequency and effective decision-making was stronger (β = −0.353, *p* < 0.001). In contrast, among footballers receiving sports psychologist support, this negative effect was significantly attenuated (β = −0.142, *p* = 0.004). These findings confirmed that psychological support functions as a cognitive buffer, mitigating the adverse effects of automatic thoughts and preserving decision-making performance under pressure.

These results support the notion that sports psychologist support serves a regulatory function in reducing cognitive load in football decision-making processes. Psychological counseling support can be considered a critical protective factor, particularly in managing negative thought patterns (Andersen, 2000; Arvinen-Barrow & Walker, 2013; Vealey & Chase, 2016).

Figure 3 illustrates that, as footballers’ automatic thought frequency increases, their levels of effective decision-making decrease. However, this negative relationship was weaker among the athletes with higher levels of psychological support. This pattern demonstrates that access to psychological counseling moderates this relationship (Dawson, 2014; Netemeyer et al., 2002). This finding suggests that psychological support helps reduce cognitive load while maintaining the quality of decisions. Therefore, psychological support plays a critical role in maintaining cognitive flexibility in footballers.

## 4. Discussion

The purpose of this research was to examine the direct, indirect, and moderating relationships between automatic thought frequency, dysfunctional attitudes, and effective decision-making in young adult footballers. The findings supported all the hypotheses; accordingly, each finding was evaluated considering theoretical foundations and similar or contrasting empirical results in the literature. The results provide strong empirical support for Beck’s cognitive model (A. T. Beck, 1995) in a sports context, confirming that footballers’ internal cognitive patterns are significant predictors of their decision-making performance. Correlation analyses showed that automatic thought frequency was positively associated with dysfunctional attitudes and negatively associated with effective decision making in sports. This result supports the theoretical framework that cognitive schemas reinforced by negative thought patterns can impair decision-making performance (Weissman & Beck, 1978). This finding also aligns with the literature demonstrating that cognitive processes in sports directly contribute to performance (Clemente et al., 2020; Raab, 2012).

The research findings indicated that as the automatic thought frequency increased, the level of effective decision-making decreased. These findings are significant when evaluated within the framework of Beck’s Cognitive Theory (A. T. Beck, 1995). This theory suggests that people’s automatic thoughts, which are often negative, lead to distortions in how they process information. In situations requiring rapid and effective decision making in sports, such distortions can adversely affect decision quality (Gökdağ & Kaçar-Başaran, 2019). Similarly, Clemente et al. (2020) demonstrated that, as cognitive load increases in footballers, decision-making accuracy decreases. In the present study, this relationship was found to be moderate (r = −0.273), indicating that automatic thoughts exert a limited but significant negative effect on decision making. This suggests that intense automatic thought activities can disrupt an athlete’s attention and reaction time in the field. These finding parallels that of Shearer et al. (2020), who demonstrated the negative effect of mental distractors on decision-making. Additionally, Parkin and Walsh (2017) reported that decision-making processes in elite athletes under physical pressure differed according to contextual factors.

This study demonstrated a significant positive relationship between automatic thought frequency and dysfunctional attitudes. This finding supports the assertion emphasized in Beck’s cognitive theory (A. T. Beck, 1995) that core schemas are reinforced by frequently repeated automatic thoughts. Automatic thoughts can activate an individual’s negative core beliefs about the self, others, and world, leading to the strengthening of cognitive biases. In this study, a moderately positive relationship (r = 0.422) was found between the two variables, and Hypothesis 2 was confirmed. Weissman and Beck’s (1978) dysfunctional attitude theory provides a theoretical explanation for this process. Similarly, Batmaz and Özdel (2016) reported that automatic thoughts strengthen dysfunctional belief systems in university students. In sports, particularly in high-pressure environments, repeated negative scenarios can activate these cognitive schemas, weakening error tolerance and self-efficacy perceptions (Gucciardi et al., 2015).

The finding that dysfunctional attitudes are negatively related to effective decision-making levels demonstrates that decision-making processes are shaped by not only momentary attention but also deep cognitive foundations. This finding aligns with Kahneman’s (2011) dual-process theory; dysfunctional attitudes may hijack cognitive resources, forcing a reliance on intuitive, error-prone ‘System 1’ thinking rather than the more analytical ‘System 2’ process. When dysfunctional attitudes suppress the analytical process of System 2, athletes may make more superficial and intuitive decisions. Oudejans et al. (2011) indicated that athletes’ decision-making skills under stress were affected by cognitive load and distorted beliefs. Vealey and Chase (2016) demonstrated that negative attitudes toward performance slow down the decision-making mechanism. In this study, the low-level negative relationship between the two variables (r = −0.205) confirms Hypothesis 3.

This study’s significant theoretical contribution is the discovery that dysfunctional attitudes partially mediate this relationship. This mediation effect was accepted as valid according to the statistical criteria for mediation models specified by MacKinnon et al. (2007). In other words, automatic thoughts frequently experienced by footballers lead to decreased decision quality, primarily by activating dysfunctional cognitive schemas rather than directly affecting decision-making ability. The calculated indirect effect coefficient was −0.043, accounting for 16.8% of the total relationships. This result supports Hypothesis 4 and demonstrates that dysfunctional attitudes play a partial mediating role. Similarly, Lemyre et al. (2007) reported negative psychological outcomes through indirect pathways in the relationship between the training load and automatic thoughts. Therefore, this finding points to the necessity of evaluating mental processes in a multi-layered manner and contributes to the understanding of the indirect relationships between mental exhaustion and decision-making in athletes.

Moderation analysis results revealed that sports psychologist support significantly altered the relationship between automatic thought frequency and effective decision making. This interaction was significant (β = 0.312, *p* = 0.002; ΔR^2^ = 0.06, *p* < 0.01) and demonstrated that among footballers receiving psychological support, the negative effect of automatic thoughts on decision-making was weaker. This result supports Hypothesis 5 and indicates that psychological support mechanisms assume protective and regulatory functions in athletes. Arvinen-Barrow and Walker (2013) stated that support from sports psychologists increases cognitive resilience in athletes and reduces the negative effects of automatic thoughts. Similarly, Thelwell et al. (2008) reported in their intervention study that athletes receiving psychological counseling support showed significant improvements in decision-making accuracy. This positions sports psychologists support not merely as a remedial intervention but also as a proactive performance-enhancement tool that builds cognitive resilience (Andersen, 2000).

### Limitations

This study has several limitations that should be acknowledged. First, the cross-sectional design does not allow for the establishment of causality between the variables; it only indicates associations. Future longitudinal studies are needed to explore the temporal dynamics of these cognitive processes and how they evolve over a season or career. Second, the reliance on a convenience sampling method, while practical for accessing a diverse group of athletes, may limit the generalizability of the findings to the entire population of young footballers. Finally, as noted in the methods section, the measure of psychological support was a binary “yes/no” variable. This approach does not capture the nuances of the intervention, such as its content, frequency, quality, or the athlete’s engagement with the psychologist. Future research should employ more detailed and objective measures to understand the specific components of effective psychological support that serve as a cognitive buffer.

## 5. Conclusions

The findings demonstrate that automatic thoughts and dysfunctional cognitive structures negatively affect decision-making performance in footballers, but this negative effect can be reduced through moderating factors, such as sports psychologist support. This research emphasizes that receiving sports psychologist support represents a critical investment in young football performance-oriented cognitive processes.

### 5.1. Recommendations for Practitioners

Coaches should consistently track the technical and tactical progress of football players and their cognitive and emotional development. Accordingly, it is essential to establish systematic and sustainable frameworks that provide athletes with the psychological support they require (Vealey & Chase, 2016). In circumstances involving competition-related stress, performance pressure, or cognitive fatigue, cognitive–behavioral intervention programs that enhance athletes’ awareness of automatic thoughts should be implemented (J. S. Beck, 2011). Embedding sports psychologists as integral members of the coaching staff plays a vital role in developing higher-order cognitive abilities, such as decision-making. Furthermore, institutional efforts to promote collaboration between coaches and psychologists can strengthen athletic performance and cognitive resilience.

### 5.2. Recommendations for Future Research

Comparative studies conducted across different age groups and sports disciplines would strengthen the generalizability of this model. Additionally, the temporal effects of automatic thought and attitude development should be tracked using a longitudinal design (Hayes, 2017). Future studies should utilize experimental methodologies to determine whether interventions, such as cognitive–behavioral training, can alter these cognitive frameworks and lead to causal improvements in decision making. Testing similar models in different cultural contexts enhances the universal validity of the findings.

## Figures and Tables

**Table 1:** Normality test results for measurement instruments.

	Kolmogorov–Smirnov			Shapiro–Wilk		
	Statistic	df	*p*	Statistic	df	*p*
Dysfunctional Attitudes	0.136	636	*p* < 0.001	0.927	636	*p* < 0.001
Automatic Thought Frequency	0.083	636	*p* < 0.001	0.973	636	*p* < 0.001
Effective Decision-Making in Sport	0.091	636	*p* < 0.001	0.957	636	*p* < 0.001

**Table 2:** Skewness statistics for measurement instruments.

Variable	N	$\bar{x}$	Skewness	SE	Std. Z
Dysfunctional Attitudes	636	2.51	0.878	0.97	0.905
Automatic Thought Frequency	636	2.18	0.239	0.97	0.246
Effective Decision-Making in Sport	636	4.04	−0.510	0.97	−0.525

**Table 3:** Confirmatory factor analysis results for data collection instruments.

	CMIN/DF (x^2^/df)	CFI	TLI	SRMR	RMSEA
Dysfunctional Attitudes	403/164 = 2.45	0.89	0.88	0.05	0.08
Automatic Thought Frequency	147/40 = 3.67	0.93	0.90	0.06	0.08
Effective Decision-Making in Sport	44/14 = 3.14	0.92	0.89	0.04	0.07

**Table 4:** Correlation analysis results for factors.

Variable	$\bar{x}$	SD	1	2	3	Cronbach’s Alpha (α)
1. Dysfunctional Attitudes	2.51	1.14	1	0.422 **	−0.205 **	0.90
2. Automatic Thought Frequency	2.18	0.73		1	−0.273 **	0.80
3. Effective Decision-Making in Sport	4.04	0.68			1	0.87

Note. N = 636. The Cronbach’s alpha coefficients are shown in parentheses on the diagonal. ** *p* < 0.01.

**Table 5:** Mediation Effect Analysis for the Model.

	Label	Estimate	SE	Z	*p*	Mediation Effect (%)
Auto_Thoughts → Dysfunctional_Attitude	a	0.659	0.05	11.75	<0.001	
Dysfunctional_Attitude → Effective_Dec_Making	b	−0.064	0.02	−2.59	0.010	
Auto_Thoughts → Effective_Dec_Making	c	−0.211	0.03	−5.43	<0.001	
Indirect	a × b	−0.043	0.01	−2.53	0.011	16.8
Direct	c	−0.211	0.03	−5.43	<0.001	83.2
Total	c + a × b	−0.254	0.03	−7.17	<0.001	100.0

**Table 6:** Cohen’s f^2^ effect size values for dependent variables in the structural model.

Dependent Variable	Predictor(s)	R^2^	Cohen’s f^2^	Effect Size
Dysfunctional Attitudes	Automatic Thought Frequency	0.18	0.22	Medium
Effective Decision-Making in Sport	Dysfunctional Attitudes, Automatic Thought Frequency	0.23	0.29	Large

**Table 7:** Moderating effect according to footballers’ receipt of sports psychologist support.

	Estimate	SE	Z	*p*
Automatic Thought Frequency	−0.248	0.0351	−7.06	<0.001
Psychologist Support	−0.182	0.0762	−2.39	0.017
Automatic Thought × Psychologist Support	0.312	0.1001	3.12	0.002
Average	−0.248	0.0353	−7.01	<0.001
Low (−1SD)	−0.353	0.0480	−7.35	<0.001
High (+1SD)	−0.142	0.0498	−2.86	0.004

Note. This table shows how the effect of automatic thought frequency (predictor variable) on effective decision making in sports (dependent variable) varies according to the level of sports psychologist support (moderator variable: yes received—no not received).

## Data Availability

The data presented in this study are available upon request from the corresponding authors. The data are not publicly available due to privacy restrictions involving participant consent.
